# Enhancing diagnosis of Hirschsprung’s disease using deep learning from histological sections of post pull-through specimens: preliminary results

**DOI:** 10.1007/s00383-023-05590-z

**Published:** 2023-11-29

**Authors:** Miriam Duci, Alessia Magoni, Luisa Santoro, Angelo Paolo Dei Tos, Piergiorgio Gamba, Francesca Uccheddu, Francesco Fascetti-Leon

**Affiliations:** 1 Division of Pediatric Surgery, Department of Women’s and Children’s Health, University of Padova, Via Giustiniani 2, 35128 Padova, Italy; 2 Pediatric Surgery Unit, Division of Women’s and Children’s Health, Padova University Hospital, Padova, Italy; 3 Department of Industrial Engineering, Padova University, Padova, Italy; 4 Surgical Pathology and Cytopathology Unit, Department of Medicine, Padova University, Padova, Italy

**Keywords:** Artificial intelligence, Hirschsprung disease, Histology, Diagnosis

## Abstract

**Purpose:**

Accurate histological diagnosis in Hirschsprung disease (HD) is challenging, due to its complexity and potential for errors. In this study, we present an artificial intelligence (AI)-based method designed to identify ganglionic cells and hypertrophic nerves in HD histology.

**Methods:**

Formalin-fixed samples were used and an expert pathologist and a surgeon annotated these slides on a web-based platform, identifying ganglionic cells and nerves. Images were partitioned into square sections, augmented through data manipulation techniques and used to develop two distinct U-net models: one for detecting ganglionic cells and normal nerves; the other to recognise hypertrophic nerves.

**Results:**

The study included 108 annotated samples, resulting in 19,600 images after data augmentation and manually segmentation. Subsequently, 17,655 slides without target elements were excluded. The algorithm was trained using 1945 slides (930 for model 1 and 1015 for model 2) with 1556 slides used for training the supervised network and 389 for validation. The accuracy of model 1 was found to be 92.32%, while model 2 achieved an accuracy of 91.5%.

**Conclusion:**

The AI-based U-net technique demonstrates robustness in detecting ganglion cells and nerves in HD. The deep learning approach has the potential to standardise and streamline HD diagnosis, benefiting patients and aiding in training of pathologists.

**Supplementary Information:**

The online version contains supplementary material available at 10.1007/s00383-023-05590-z.

## Purpose

Hirschsprung disease (HD) is a congenital disease characterised by the absence of ganglion cells in the distal bowel, extending proximally for varying distances [1]. A variety of diagnostic tests including contrast enema and anorectal manometry may be used as diagnostic screens, but diagnosis ultimately lays upon histopathological examination of a rectal biopsy. The diagnostic histological features of HD include the absence of ganglion cells and an increase in hypertrophic cholinergic nerves [2]. The accurate assessment of these histological features plays a pivotal role in planning a correct surgery needed to remove the non-functioning bowel. However, this histological analysis is far from straightforward. It presents several challenges, primarily in the accurate differentiation of these structures and is susceptible to errors due to the potential for variations in interpretation, which can significantly impact the diagnosis and consequently, treatment decisions. To address these challenges, artificial intelligence (AI) has emerged as a valuable tool in pathology [3, 4]. AI algorithms offer the promise of delivering consistent results, mitigating the interobserver variability that often can occur in manual assessment, thus facilitating the comparison of cases across different medical institutions [3, 5]. Furthermore, especially in low-volume centres, AI can help offset the deficiency in specialised expertise, thereby enhancing the quality of care provided to HD patients. In this study, we introduce an AI-based method aimed at automating the identification and quantification of ganglionic cells and hypertrophic nerves in the resected specimens for HD.

## Materials and methods

### Participant and slide selections

Specimens collected from patients undergoing surgical treatment for Hirschsprung’s disease (HD) between January 2010 and January 2022 were included in this study. Slides with poor quality, such as those exhibiting drying effects, significant air bubbles, or broken glass, were excluded from the analysis. Only formalin-fixed, paraffin-embedded tissue samples stained with haematoxylin–eosin were considered for the study. A high-capacity scanner was employed to capture images of 2048X1280 pixels at X10 magnification, thereby generating the dataset.

### Development and training of AI system

The proposed methodology consisted in different steps:

1. Annotation: A dedicated team comprising an expert pathologist and a paediatric surgeon specialising in HD histology meticulously annotated high-resolution slides (2048X1280 pixels at X10 magnification) using the AAPER web-based platform. Their meticulous work involved identifying and encircling ganglionic cells, nerves, and hypertrophic nerves individually. The annotations made on each image were then exported as multiclass masks. This process resulted in the creation of a tiff file for each histological slide, ultimately yielding two datasets: one for the histological images and another for the corresponding masks.
2. Data augmentation: To bolster the training process and mitigate the risk of overfitting, we applied data augmentation techniques. These techniques encompassed random flipping, rotation, and colour normalisation.
3. Dataset subdivision: The entire dataset underwent division into two subsets. The first subset, constituting 80% of the entire dataset, served as the training set for algorithm and model development. Simultaneously, the second subset, encompassing 20% of the dataset, was reserved for subsequent analysis and validation purposes.
4. Manual segmentation: each entire slide was partitioned into 40 squared patches, each measuring 256 × 256 pixels. This division not only enhanced the overall output quality but also further increased the volume of data available for analysis.
5. Train supervised U convolutional neural network: This extensive dataset served as the foundation for training neural networks. To create various neural network models, we curated subsets of the dataset, each with specific image characteristics. One dataset exclusively contained images of ganglion cells and normal nerves, while another comprised solely hypertrophic nerves. To ensure comprehensive learning, we also introduced images characterised solely by background into both datasets. This approach enabled the network to not only recognise the target areas for classification but also understand the broader context of the images, where normal cells and nerves would never appear, unlike hypertrophic nerves. In summary, the datasets we generated encompassed images with the following characteristics:

Dataset 1—model 1: Ganglionic cells and normal nerves

Dataset 2—model 2: Hypertrophic nerves:

The expected segmentations when applying model 1 are as follows

For images within the ganglionic zone: Segmentation of normal nerves and ganglionic cells

For images within the transition zone: Segmentation of ganglionic cells

For images within the aganglionic zone: No segmentation

For images containing only background: No segmentation

When applying model 2, the anticipated segmentations are:

For images within the ganglionic zone: No segmentation

For images within the transition zone: Segmentation of hypertrophic nerves.

For images within the aganglionic zone: Segmentation of hypertrophic nerves.

For images containing only background: No segmentation.

**Fig. 1:**
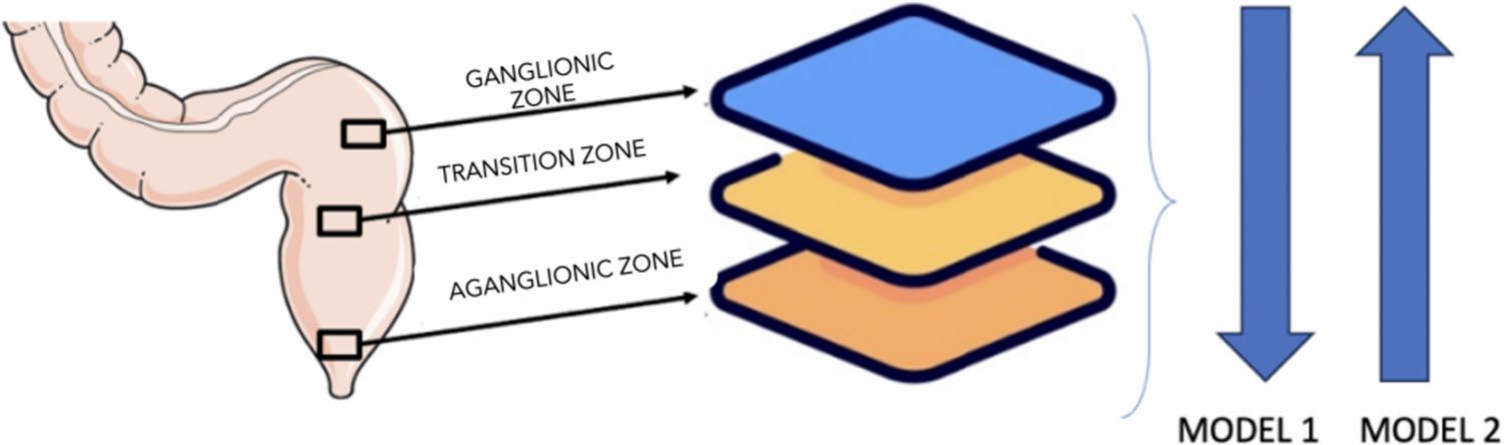
Description of AI models

Consequently, there are images segmented by both model 1 and model 2, all of which belong to the transition zone and require additional scrutiny by the pathologist (Fig. 1).

6. Testing convolutional neural network: Some slides were used to test the proposed model. In this context, each whole image of 2048X1280 pixels was divided into 40 patches with a size of 256 × 256 pixels. The predict function was then applied to each patch, and subsequently, the patches were reassembled using the un-patchify function to generate the original-sized mask. The manually segmented masks served as a verification control.

### Analysis

At the conclusion of the training process, the evaluation function was applied to the model, yielding a metric value. In our study, we assessed accuracy, which provides insight into the proximity of predicted values to their true counterparts. This metric was determined by dividing the number of correct predictions by the total number of predictions made (Accuracy = correct prediction/all prediction). Achieving a high accuracy, typically exceeding 90%, is indicative of strong model performance. In addition to evaluating the model's ability to perform automatic segmentation, a prediction function was employed for each input sample, using this mathematical function: y_pred = model.predict(x_test). In our case, the input consists of the X test dataset, comprising images from the validation set representing 20% of the original dataset. This dataset was also used to assess the model’s accuracy at the end of each epoch.

## Results

During the study period, we identified a total of 31 eligible patients diagnosed with Hirschsprung’s disease (HD). From these patients, we collected a comprehensive set of 108 tissue samples representing various regions, including the ganglionic zone, transition zone, and aganglionic zone. All these samples were preserved, paraffin-embedded, and formalin-fixed, and they were stored in the archive of the Institute of Pathology at a single specialised referral centre. Ganglionic cells, nerves, and hypertrophic nerves were annotated using a web-based platform (AAPER). The annotations were then used to generate masks, which would serve as essential inputs for the neural network. Following this, we took steps to augment the dataset to obtain 540 slides. Out of these, 50 were excluded from the training process as they were reserved for testing the U-NET model. Considering 490 slides, manual segmentation was applied to yield an extensive dataset consisting of 19,600 images. However, during the data processing phase, we excluded 17,655 slides that did not contain the specific target elements we were interested in. Subsequently, the AI algorithm was trained using the remaining 1,945 slides (930 for model 1 and 1015 for model 2), setting the stage for our comprehensive analysis and evaluation.

### Model 1: Ganglionic cells and normal nerves

**Fig. 2:**
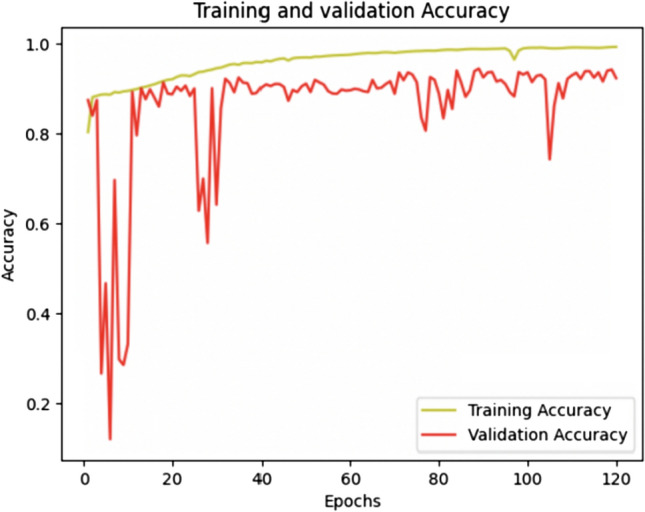
Training and validation accuracy graph of model 1

**Fig. 3:**
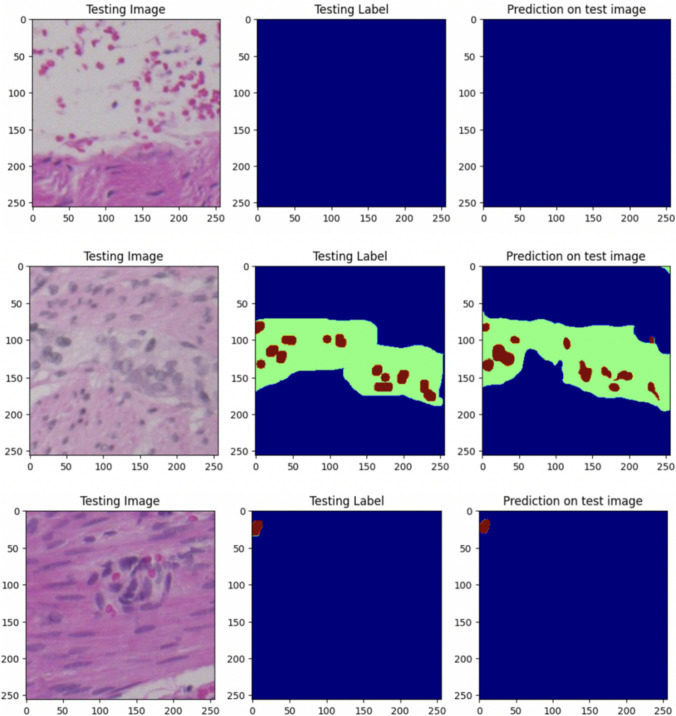
Model 1: “Testing images” are the images belonging to the validation set; “Testing label” refers to the corresponding manually segmented masks provided by the pathologist, and “prediction on test image” pertains to the predictions generated by the model. Red = ganglionic cells. Green = normal nerves. Blue = background

Three classes were considered for this segmentation: ganglionic cells, normal nerves, and background. The initial dataset comprised 930 images, divided as follows: 744 images for the training set and 186 images for the validation set. The training involved 120 epochs, and as demonstrated in Fig. 2, this training was deemed sufficient as a plateau was reached starting from the 100 epochs (green curve). Applying this scheme, the diagnostic accuracy reached 92.3% (Fig. 2—red curve). Figure 3 describes the prediction model related to the model 1.

### Model 2: hypertrophic nerves

**Fig. 4:**
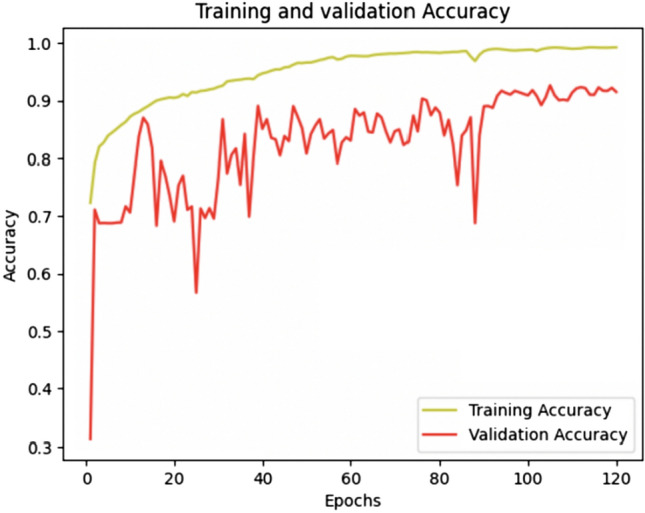
Training and validation accuracy graph of model 2

**Fig. 5:**
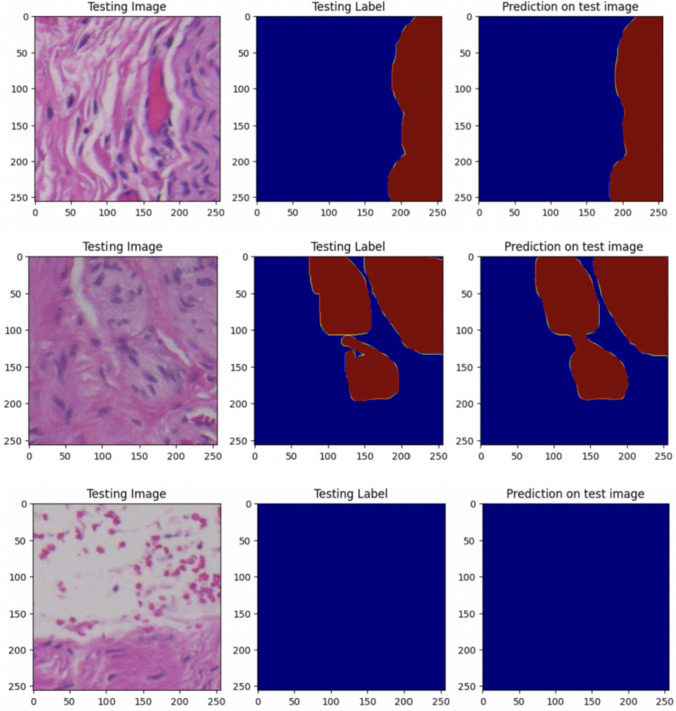
Model 2: “Testing images” are the images belonging to the validation set; “Testing label” refers to the corresponding manually segmented masks provided by the pathologist, and “prediction on test image” pertains to the predictions generated by the model. Bordeaux = hypertrophic nerves. Blue = background

For training this model, we focussed on just two classes: hypertrophic nerves and background. The initial dataset comprised 1015 images, with the following distribution (812 images for the training set; 203 images for the validation set). Training involved 120 epochs, and as depicted in Fig. 4, the training accuracy reached a plateau starting from the 98th epoch, indicating a sufficient number of epochs for effective training. The calculated accuracy value of this model was 91.50% (Fig. 4)*.* As for prediction, Fig. 5 demonstrates the model’s proficiency in accurately identifying the presence of both classes.

### Test set evaluation

During this phase, we employed the 50 slides that were excluded from the training set and applied both models 1 and 2, which had undergone validation in previous stages. When model 1, designed to identify ganglionic cells and normal nerves, was applied to images from the ganglionic zone, it detected a higher number of ganglionic cells than were actually present (Supplementary Fig. 1). In the case of images from the aganglionic zone, when model 1 was employed, only 4 out of 40 patches were incorrectly segmented (Supplementary Fig. 2). On the other hand, applying model 2, specifically trained to recognise hypertrophic nerves, to ganglionic region images resulted in only 3 out of 40 patches being misclassified (Supplementary Fig. 3). When model 2 was applied to ganglionic regions, the system accurately segmented them (Supplementary Fig. 4).

## Discussion

Artificial intelligence (AI) and machine learning are emerging technologies that can be used to create algorithms capable of decision making [6]. The whole medical scientific community has been fascinated by this new opportunity. Researchers and clinicians dedicated to rare conditions are foreseeing tools which would overcome the scarcity of numerosity of existing series to reach robust and supported diagnostic and therapeutic processes. Furthermore, the specific field of diagnosis through images both from radiology exams and pathology specimens would clearly receive huge support from AI-based assessments [7, 8]. Digital pathology, coupled with advanced digital slide scanning technology, has opened numerous possibilities for identifying various tissue types and specific target elements [9, 10]. Through the application of machine and deep learning techniques, it is now feasible to train a “computer pathologist” to recognise diverse structures, depending on their unique characteristics. However, one current limitation of fully automated pathology lies in the need for pathologist-guided delineation of specific regions within digitised slides. To achieve a diagnostically conclusive result, it has become increasingly important to blend both manual-adapted detection and automated cellular analysis through deep learning methods. Wang et al. have highlighted the advantages of combining these approaches to mitigate issues arising from the vast amount of data or a lack of inherent understanding of histological structures [12]. Deep learning relies on extensive datasets to train neural network algorithms [11]. As the number of slides/images increases, the algorithm's capability for unsupervised cellular analysis improves, enabling it to recognise disease-specific features and patterns through learned associations [12]. Until recently, AI and machine learning technologies were predominantly applied in the field of oncology [13–15]. However, more recently, these systems have been introduced into the diagnostic process of rare paediatric diseases, holding great promise [16–19]. Hirschsprung disease is a rare condition, belonging to the anomalies of the enteric nervous system. The condition is diffused all over the world with an incidence of 1 out 5000 newborns [20]. Although several aspects of the disease have been deeply studied, aetiology as well as variability in the phenotype and prognosis are still challenging the specialists who treat it. Guidelines for diagnosing and treating these cases are emerging from the editorial effort of medical societies and supranational institutions with methodology conditioned by poor level of evidence [21, 22]. In Hirschsprung’s disease and allied disorders, the expert’s involvement in crucial phases is rewarded as the possible guarantee of a correct approach. However, expertise definition is currently vague and volume of treated cases seems the only reliable parameter.

In this study, we have described the development and technical validation of a novel, supervised AI model for the evaluation of histopathologic features in the spectrum of Hirschsprung diagnosis. The primary goal of this study was to establish a “proof-of-principle” model in the setting of HD-AI system, showing its potential as a semi-automated tool in the field of anatomic pathology, providing accurate, reproducible, quantitative assessment of various microscopic features of interest (identifying ganglionic cells, hypertrophic nerves, normal nerves), increasing both efficiency and reporting standardisation in this specific context. Considering the rare nature of Hirschsprung's disease, there have been limited attempts to harness AI for its diagnosis. Schilling et al. made an endeavour in this direction, utilising AI to diagnose HD with the aid of histological slides stained for calretinin, microtubule-associated protein 2, Glucose transporter isoform 1, and S100. Their study involved 93 tissue blocks from 31 specimens of 27 patients. In their training set, they reported a sensitivity of 87.5% and specificity of 80%, while in the development set, they achieved 95% sensitivity and 90.4% specificity [18]. Our study diverges in both objectives and methodologies. First, mirroring a recent study by Greenberg et al., we exclusively employed H&E-stained slides, abstaining from the use of immunohistochemistry [23]. Second, through the diligent application of data augmentation and segmentation techniques, our dataset surpassed 1000 slides in volume, distinguishing it in terms of scale and potential.

The algorithm developed in this study demonstrates an accuracy rate of 92.3% for detecting ganglionic cells and 91.5% for identifying hypertrophic nerves, respectively. In the realm of Hirschsprung’s disease diagnosis, a common trend in the literature is the consistently high reported specificity, typically exceeding 90%, which translates to a rarity of false-positive results. However, the incidence of false-negative results displays a wider spectrum, ranging from 0 to 40% [24]. In this context, the potential incorporation of immunohistochemistry could further enhance diagnostic accuracy. Nevertheless, it is noteworthy that at our centre, our experienced pathologist achieved a 100% detection rate for pathological markers (including hypertrophic nerves and the absence of ganglionic cells) exclusively through the examination of H&E-stained slides, without any instances of false positives. Consequently, the utilisation of this algorithm may simplify the diagnostic process and empower less-experienced pathologists to perform effectively. To the best of our knowledge, this study represents the pioneering effort to employ two AI models for the histological diagnosis of Hirschsprung’s disease, encompassing both ganglionic cells and hypertrophic nerves. This innovation is significant because the combined use of these models enables the AI system to identify the transition zone, the area situated between the aganglionic and ganglionic zones. Notably, our research group has previously demonstrated that the length of this transitional area serves as a predictive factor for post-HAEC development (these findings are yet to be published). One of the most significant challenges encountered in this model is the accurate detection of ganglion cells. The machine learning algorithm may occasionally misclassify immature ganglion cells as mature ganglion cells, especially when they are not in proximity to the expected context. It is essential to note that ganglion cells are exclusively located within the submucosa or muscularis propria layers. Any cell or finding identified in any other layer, regardless of how similar it may appear, is highly unlikely to represent a genuine ganglion cell. However, in the absence of this contextual information, some findings can mimic ganglion cells, particularly immature ones, leading to potential misclassification.

To address this issue, our future applications of the algorithm will include tracking the origin of each image within its respective slide. This additional contextual information will significantly enhance the algorithm’s ability to provide a more accurate assessment by considering the specific histological layer in which the cells are located.

This study has several limitations which merit mention. As stated, the available dataset was limited and significantly smaller than that of similar studies on the use of AI in pathology [25, 26]. Large data sets are considered necessary to properly represent the wide variability present in clinical samples. Smaller datasets therefore suffer both from a statistical standpoint and from excessive uniformity. Our use of data augmentation techniques somewhat circumvents this problem. Nevertheless, additional data, including data generated by other institutions, considering the rarity of the disease would allow for further validation which could improve upon the algorithm. Furthermore, from a technical point of view, there are various challenges that will have to be overcome. For instance, artefacts can be mistaken as ganglionic cells if many tissue layers overlap and create a “brown-like colouration”. In addition, in a machine learning approach for histological purposes, the hierarchical analysis of specific structures such as nerves and ganglion cells within the tissue slide is a fundamental aspect that significantly contributes to the accuracy and effectiveness of the AI system.

## Conclusion

The results demonstrate the robustness of the AI- based U-net technique in accurately detecting ganglion cells and nerves in HD histology. Furthermore, the streamlined nature of AI-based diagnosis can significantly benefit patients. Timely and accurate diagnoses are crucial in HD, as they directly impact the planning post-operative care. By reducing the time required for histological analysis, we can expedite faster treatment decisions and improve patient outcomes. The increase in data transfer speed associated with may predict scenarios where an AI-based pathology assistant may indicate if and where to transfer the case for a definitive diagnosis made by an human expert. Moreover, the integration of AI can also play a role in the training of pathologists. The technology serves as a valuable educational tool, allowing pathologists with special interest towards rare conditions, to learn from a vast dataset of annotated cases.

## Supplementary Information

Below is the link to the electronic supplementary material.

Supplementary file1 (PDF 942 KB)

## Data Availability

Requests for data sharing will be considered upon written request to the corresponding author.
