# Supplementary material for: Enhancing diagnosis of Hirschsprung’s disease using deep learning from histological sections of post pull-through specimens: preliminary results
Source: Pediatr Surg Int. 2023 Nov 29;40(1):12. doi: 10.1007/s00383-023-05590-z (PMC10687181; doi:10.1007/s00383-023-05590-z)
Supplement: Supplementary file 1 — Supplementary file1 (PDF 942 KB) [file 383_2023_5590_MOESM1_ESM.pdf]

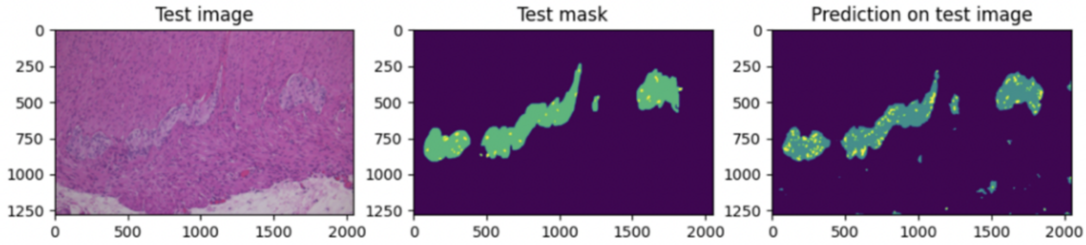

Supplementary fig 1: “Testing images” are the images belonging to the testing set of the ganglionic area; “Test mask” refers to the corresponding manually segmented masks provided by the pathologist that were used to verify the automatic prediction and “prediction on test image” pertains to the predictions generated automatically by the model 1.

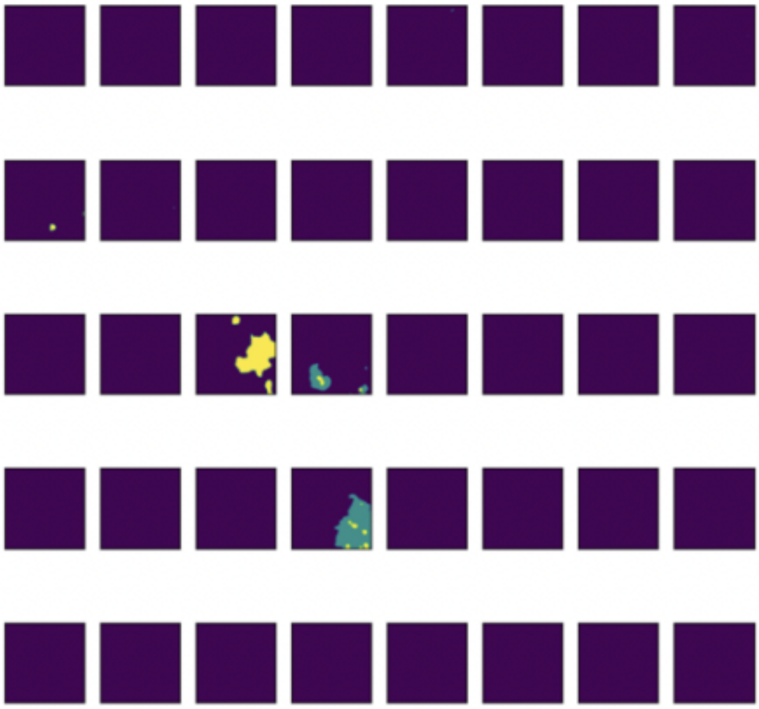

Supplementary fig 2: Automated segmentation of the aganglionic region resulted in the misclassification of 4 patches, where possible normal nerves (green) and potential ganglionic cells (yellow) were erroneously identified.

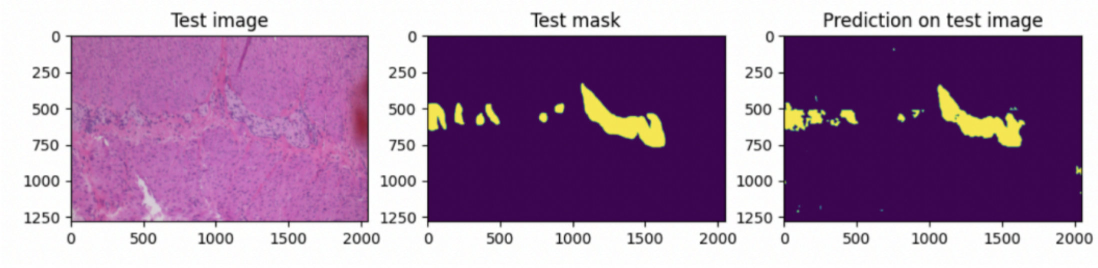

Supplementary fig 3: “Testing images” are the images belonging to the testing set of the aganglionic area; “Test mask” refers to the corresponding manually segmented masks provided by the pathologist that were used to verify the automatic prediction and “prediction on test image” pertains to the predictions generated automatically by the model 2.

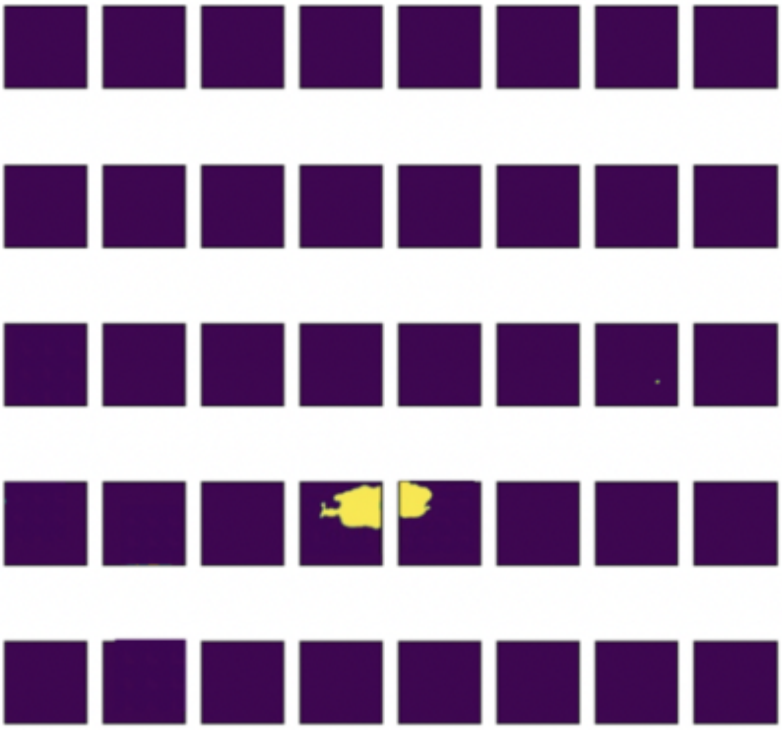

Supplementary fig 4: Automated segmentation of the aganglionic region resulted in the misclassification of 2 patches, where possible hypertrophic nerves (yellow) were erroneously identified.
